# The findings of optical coherence tomography of retinal degeneration in relation to the morphological and electroretinographic features in *RPE65*^−/−^ mice

**DOI:** 10.1371/journal.pone.0210439

**Published:** 2019-01-29

**Authors:** Reiko Tanabu, Kota Sato, Natsuki Monai, Kodai Yamauchi, Takayuki Gonome, Yuting Xie, Shizuka Takahashi, Sei-ichi Ishiguro, Mitsuru Nakazawa

**Affiliations:** 1 Department of Ophthalmology, Hirosaki University Graduate School of Medicine, Hirosaki, Japan; 2 Department of Ophthalmology, Tohoku University Graduate School of Medicine, Sendai, Japan; Justus Liebig Universitat Giessen, GERMANY

## Abstract

**Purpose:**

Mutations of the gene encoding RPE65 cause Leber congenital amaurosis (LCA) retinitis pigmentosa (RP). The optical coherence tomography (OCT) is increasingly utilized to noninvasively evaluate various types of retinal diseases, including RP. The present study was conducted to characterize the OCT findings of the *RPE65*^−/−^ mice—an animal model of LCA and RP—in relation to the morphological features based on histological and electron microscopic findings as well as electroretinography (ERG) features.

**Materials and methods:**

*RPE65*^−/−^ mice were employed as a model of retinal degeneration. C57BL/6J mice were used as a wild-type control. OCT was performed on the *RPE65*^−/−^ mice from postnatal day (P) 22 to 170. The longitudinal changes in the OCT images and fundus pictures were analyzed both qualitatively and quantitatively in comparison to those of C57BL/6J mice. The OCT images were also compared to the histological and electron microscopic findings. Full field combined rod and cone ERG was performed to analyze the relationship between morphology based on OCT and the amplitudes of the a- and b-waves.

**Results:**

In the *RPE65*^−/−^ mice, the photoreceptor rod and cone layer appeared as a diffuse hyperreflective zone contiguous with the inner segment ellipsoid zone (IS-EZ) on OCT, even on P22, whereas the IS-EZ and interdigitation zone were clearly identified in the age-matched C57BL/6J mice. The histological analyses revealed that the regular arrangement of the photoreceptor inner and outer segments was gradually lost in the *RPE65*^*-/-*^ mice. On electron microscopy, most of the rod outer segments were degenerated from P21 to P35, whereas outer segments became variably shorter after P49 although ultrastructure appeared to normalize. The thickness of the outer nuclear layer of *RPE65*^−/−^ mice was slowly and progressively reduced in comparison to C57BL/6J mice. Although the thickness of the inner and outer segment layer of *RPE65*^−/−^ mice was significantly decreased in comparison to C57BL/6J mice, the change was not progressive, at least until P170. Even at P35, the amplitudes of both a- and b-waves on ERG were severely deteriorated in comparison to those of C57BL/6J mice. Mottled depigmented spots appeared throughout the fundus in *RPE65*^−/−^ mice after P72, and were detected as hyperreflective deposits under the retinal pigment epithelium on OCT.

**Discussion:**

The pathological changes in the inner and outer segments layer of *RPE65*^−/−^ mice were identified as diffuse hyperreflective changes on OCT. The rod outer segments showed degeneration in the early postnatal periods but became morphologically normalized in the disc structure after P49, although the sizes of the length of the rod outer segments were variable. OCT could not qualitatively differentiate the early degeneration of rods from the late variability in size of rods. Although the morphology of the photoreceptor outer segments was relatively preserved in the *RPE65*^−/−^ mice, the amplitudes of ERG were severely disturbed. These structural and functional deficits may be derived from the defective supply of 11-cis-retinol to the photoreceptors.

## Introduction

RPE65 (retinal pigment epithelium-specific-65-kDa) is an enzyme protein that is specifically expressed in the retinal pigment epithelium (RPE) and which catalyzes the isomerohydration of all-trans-retinyl ester to 11-cis-retinol [1]. It is an essential reaction in the visual cycle that provides regenerated 11-cis-retinal as a chromophore to the rod and cone opsin apoproteins. Mutations of the gene coding for RPE65 result in the impairment of the visual cycle and cause retinal dystrophies, such as Leber congenital amaurosis (LCA) and retinitis pigmentosa (RP) [2–10]. Recently, gene therapy was performed for patients with RP or LCA associated with mutations in the *RPE65* gene [11–13] and the subsequent clinical course after gene therapy was investigated [14]. *RPE65*-knockout (*RPE65*^−/−^) mice that had been created from C57BL/6J mice [15] demonstrated slow rod degeneration and rapid cone degeneration [15–17], and can be considered to be a model of human *RPE65* gene-associated LCA and RP in humans, although there may be some differences in the course of retinal degeneration between *RPE65*^−/−^ mice and human patients with *RPE65*-associated LCA and RP [18].

Recent advances in optical coherence tomography (OCT) technology have revealed a number of previously unknown morphological details regarding the mechanisms of various retinal diseases, including LCA and RP [19–32]. Although OCT has an advantage in that it can noninvasively investigate the morphological features of LCA and RP, it is impossible to directly assess the pathological features from certain OCT images. Animal models associated with known mutations are useful in providing clues to understand the relationship between OCT images and their pathological backgrounds. Although the retinal OCT findings in animal models of LCA or RP caused by some known gene mutations, including genes associated with rhodopsin, phosphodiesterase ß-subunit, arrestin or mertk have already been reported [33–39], those of *RPE65*^−/−^ mice have not been described. It would be clinically useful to understand the pathological origins of the abnormal retinal features on OCT that are associated with *RPE65* gene mutations, particularly in LCA or RP patients with these mutations. The present study was undertaken to both qualitatively and quantitatively characterize the OCT findings of the *RPE65*^−/−^ mice. The aim of the present study was to clarify the correlation between the OCT findings and the morphological and electroretinography (ERG) findings caused by the mutation of *RPE65*. In this study, the morphological findings were based on histological and electron microscopic features.

## Materials and methods

### Experimental animals

All experimental procedures performed in this study conformed to the regulations of the Association for Research in Vision and Ophthalmology (ARVO) Statement for the Use of Animals in Ophthalmic and Vision Research, and were approved by the institutional Committee of Ethics for animal experiments (Approval Number: M12023).

The *RPE65*^−/−^ mice were generously provided by Mathew M. LaVail (University of California, San Francisco, CA, U.S.A.) and T. Michael Redmond (National Eye Institute, Bethesda, MD, U.S.A.). C57BL/6J mice were purchased from Clea, Japan (Tokyo, Japan) and were used as wild-type controls [15]. The mice were maintained at the Hirosaki University Graduate School of Medicine Animal Care Service facility under a cycle of 12 h of light (50 lx illumination) and 12 h of darkness (<10 lx environmental illumination) in an air-conditioned atmosphere. The animals had *ad libitum* access to food and water.

### OCT examination and fundus photography

**Fig 1:**
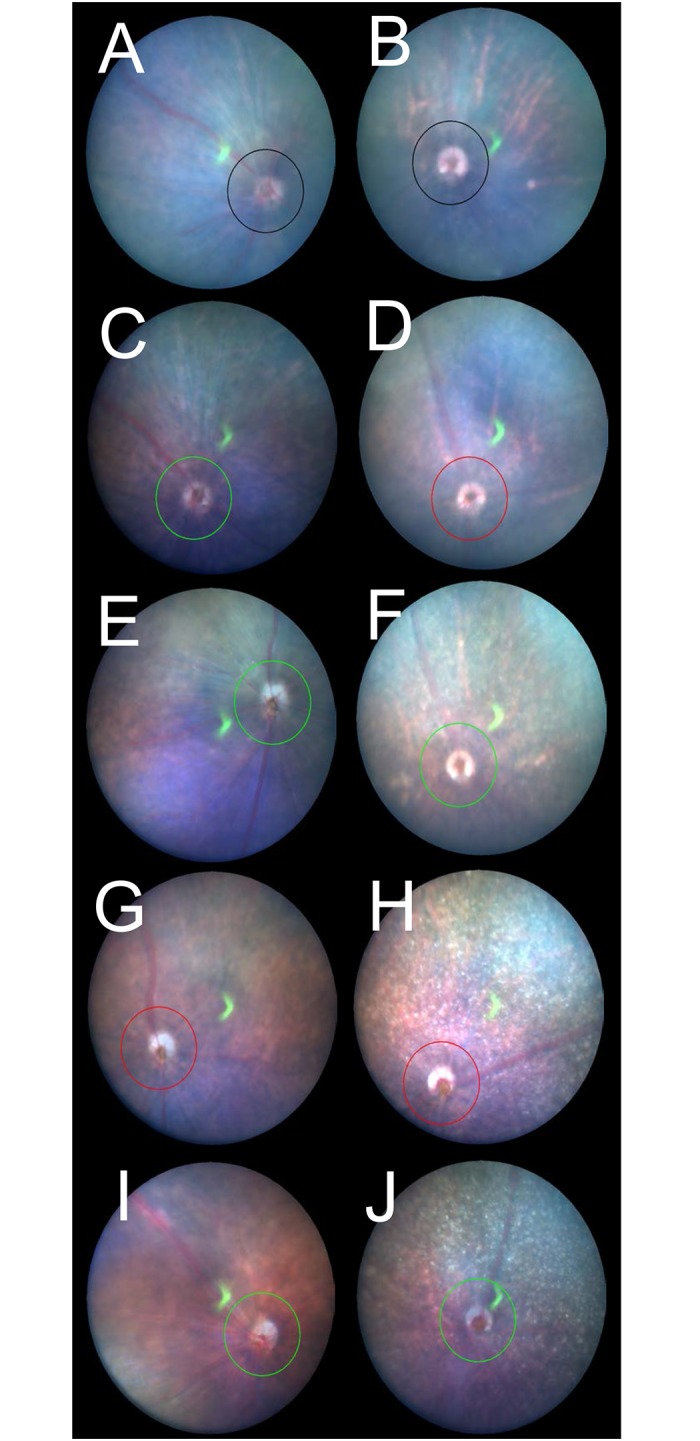
Representative fundus pictures of the C57BL/6J (A, C, E, G and I) and *RPE65*^−/−^ (B, D, F, H and J) mice. A, A C57BL/6J mouse on P22. B, An *RPE65*^−/−^ mouse on P22. C, A C57BL/6J mouse on P36. D, An *RPE65*^−/−^ mouse on P32. E, A C57BL/6J mouse on P72. F, An *RPE65*^−/−^ mouse on P79. G, A C57BL/6J mouse on P106. H, An *RPE65*^−/−^ mouse on P113. I, A C57BL/6J mouse on P169. J, An *RPE65*^−/−^ mouse on P170. Circles indicate the line at which the OCT images were created.

OCT and fundus photography were performed according to the methods previously described, using a Micron IV (Phoenix Research Labs, Pleasanton, CA, U.S.A.) [37, 39]. Briefly, OCT and fundus photography were carried out at 8 time points from postnatal (P) day 22 to P170 (P22, P27, P32, P61, P79, P113, P155, and P170) for *RPE65*^−/−^ mice and at 6 time points from P22 to P169 (P22, P36, P72, P106, P148 and P169) for C57BL/6J mice. Three or four mice were examined at a time. The mice were anesthetized with an intraperitoneal injection of a mixture of medetomidine hydrochloride (0.315mg/kg), midazolam (2.0mg/kg), and butorphanol tartrate (2.5mg/kg). The pupils were dilated with the instillation of eye drops containing a mixture of 0.5% tropicamide and 0.5% phenylephrine hydrochloride. The mouse ocular fundus was simultaneously monitored by a fundus camera, and the position of the retinal OCT image was set circumferentially around the optic disc by considering potential structural differences between the upper and lower hemispheres of the mouse eyes (Fig 1) [40]. The diameter of the circle section was 500μm and it provided the 360° section around the optic disc at 140μm from the optic disc margin. To analyze the structure of a certain fundus change of interest, the position of the OCT image was set vertically or horizontally, depending on the finding. The corneal surface was protected using a 1.5% hydroxyethylcellulose solution. Fifty images were averaged to eliminate the projection artifacts. The quantitative analysis of the acquired OCT images was performed using the InSight software program (Phoenix Research Labs). During all experimental procedures, the physical condition of the mice was frequently monitored by inspection and gentle palpation by the researchers.

### The analysis of the retinal layer thickness

We measured the thickness of the inner (A), outer nuclear (B), and photoreceptor inner segment (IS) and outer segment (OS) (C) layers of the neural retina and the combined the retinal pigment epithelium (RPE) and choroid layers (D, S1 Fig). The inner retinal layer A consisted of the retinal nerve fiber layer (NFL), the ganglion cell layer (GCL), the inner plexiform layer (IPL), the inner nuclear layer (INL) and the outer plexiform layer (OPL) (see details in S1 Fig).

Segmentation was performed using the InSight software program, as previously reported [33, 39]. The borderlines between each sublayer were automatically identified by the software program using OCT images and were manually corrected by the researchers if necessary. The average distance (μm) between each borderline was manually calculated using the raw data automatically summarized in the Excel^®^ file generated by the InSight software program. The data obtained from both eyes of the same animal were averaged. The overall average retinal layer thickness was presented as the mean ± standard deviation (S1 and S2 Tables).

### Histological examination

Histological examinations were performed using eyes enucleated from *RPE65*^−/−^ mice on P95 and P127 and C57BL/6J mice on P66 and P120. Immediately after euthanasia by luxation of the cervical spine, the eyes were excised under a microscope. To prevent the possibility of artificial retinal detachment during further processing, an aliquot of 2% glutaraldehyde and 2% paraformaldehyde solution (pH 7.4) was injected into the anterior chamber through the corneal limbus. After fixation in the same solution for 2h at room temperature, the eyeballs were re-fixed in 4% paraformaldehyde solution at pH 7.0 for 24h at 4°C. Paraffin embedding, sectioning, and hematoxylin and eosin (HE) staining were performed as previously described [33, 39]. The HE-stained sections were photographed under a light microscope (DP-71, Olympus, Tokyo, Japan). The histological findings were compared to the corresponding findings from OCT images.

### Electron microscopy

Electron microscopy was performed using eyes enucleated from *RPE65*^−/−^ mice on P21, P35, P49, P95, and P127 and from C57BL/6J mice on P21, P66, and P120 according to a previously described method [33, 39]. Immediately after enucleation, the eyes were fixed with 2.5% glutaraldehyde and 2% paraformaldehyde solution (pH 7.4) for 24 h at 4°C. Similarly to the preparation for the histological examination, an aliquot of the same fixation solution that was used for the histological study was injected into the anterior chamber. The retina and choroid were dissected out, post-fixed in phosphate buffered 1% osmium tetroxide (pH 7.4) for 3 h at 4°C, dehydrated in an ascending ethanol series (50–100%), and embedded in epoxy resin. Thin sections (80–90 nm) were stained in uranyl and lead salt solutions. The sections were photographed by a transmission electron microscope (H-7600, Hitachi, Tokyo, Japan) at 100kV.

### Electroretinography (ERG)

Scotopic full-field combined rod and cone ERG was performed using a Micron Ganzfeld ERG system (Phoenix Research Labs) according to the manufacturer’s instructions as previously reported for rats [37, 39]. ERG was measured at 3 time points from P31 to P157 (P31, P83 and P157) for *RPE65*^*-/-*^ mice, and at 3 time points from P35 to P150 (P35, P87, P150) for C57BL/6J mice. In brief, after being dark adapted for at least 24 h, the mice were anesthetized by the same method as for the SD-OCT examination. A reference electrode was placed in the center of the scalp, and a ground electrode was set in the proximal portion of the tail skin. During the measurement, the body temperature was kept at 37°C, using a body warmer. The pupils were dilated by eye drops containing a mixture of 0.5% tropicamide and 0.5% phenylephrine hydrochloride. After the corneal surface was anesthetized using 0.4% oxybuprocaine hydrochloride eye drops, a contact-lens electrode was applied directly to the corneal surface. The light stimulus was fixed at 3.0 cd.s/m^2^, after the stimulus-dependent manner in response was confirmed by changing the stimulation from 3.0 to 30.0 cd.s/m^2^ in a group of wild-type mice according to the ICEV standard [41]. The overall average amplitudes of both a- and b-waves were presented as the mean ± standard deviation.

### Statistical analyses

The statistical analyses of the data obtained in the present study were performed using the SPSS software program (version 25, Statistical Package for the Social Sciences, Chicago, IL, U.S.A.). The segmentation data from the two groups were compared using a two-way repeated analysis of variance (two-way repeated ANOVA) after the normality and equality of each distribution were confirmed by the Shapiro-Wilk test and Levene’s test, respectively. Student’s *t*-test was performed to analyze differences in OCT segmentation between similar age-groups (*RPE65*^−/−^ vs. C57BL/6J: P22 vs. P22, P79 vs. P72, P113 vs. P106, P155 vs. P148, and P170 vs. P169, respectively). For the ERG data, the amplitudes and latencies of the a- and b-waves (*RPE65*^−/−^ vs. C57BL/6J: P31 vs. P35, P83 vs. P84, and P157 vs. P150, respectively) were statistically compared using Student’s *t*-test after confirming the normality of each distribution by the Shapiro-Wilk test. *P* values of < 0.05 were considered to indicate statistical significance.

## Results

### The fundus findings of C57BL/6J and *RPE65*^−/−^ mice

The long-term changes in the fundus findings of both C57BL/6J and *RPE65*^−/−^ mice are presented in Fig 1. In contrast to the findings in the C57BL/6J mice, the *RPE65*^−/−^ mice fundus showed diffuse depigmented spots, particularly after P79, when diffuse white spots that were different from the choroidal vascular tessellation were identified (Fig 1F, 1H and 1J). These findings appeared to correspond to the previously reported ocular findings of retinal degeneration in patients with LCA associated with compound heterozygous Leu67Arg/Tyr368Cys mutations in the RPE65 gene [42].

### The qualitative analyses of the OCT findings in relation to the photoreceptor structure in the *RPE65*^−/−^ mice

**Fig 2:**
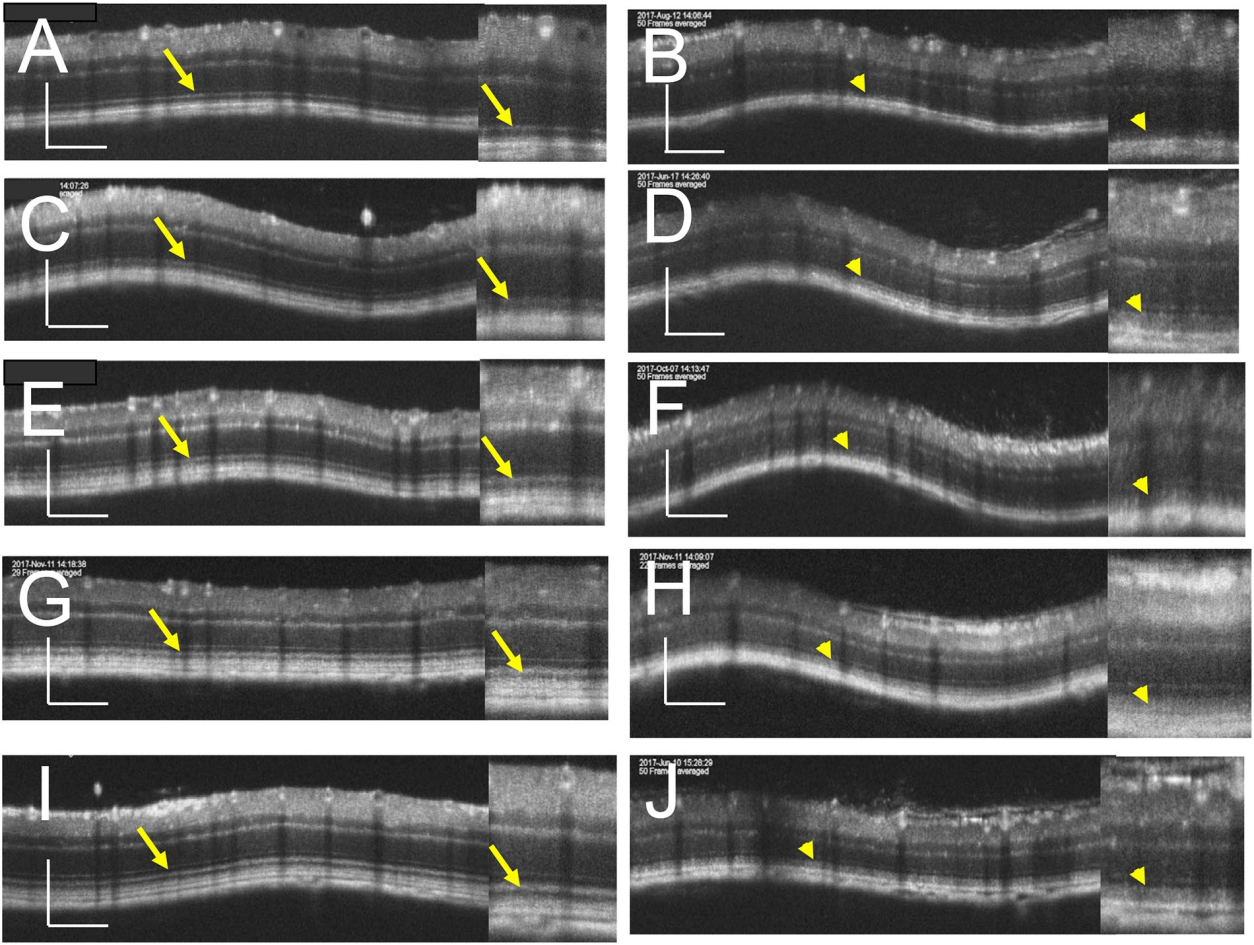
Representative OCT images of the C57BL/6J (A, C, E, G and I) and *RPE65*^−/−^ (B, D, F, H and J) mice. A, A C57BL/6J mouse on P22. B, An *RPE65*^−/−^ mouse on P22. C, A C57BL/6J mouse on P36. D, An *RPE65*^−/−^ mouse on P32. E, A C57BL/6J mouse on P72. F, An *RPE65*^−/−^ mouse on P79. G, A C57BL/6J mouse on P106. H, An *RPE65*^−/−^ mouse on P113. I, A C57BL/6J mouse on P169. J, An *RPE65*^−/−^ mouse on P170. The right panel of each picture is a magnified image. Arrows indicate the photoreceptor inner segment ellipsoid zone. Bar indicates 100μm.

**Fig 3:**
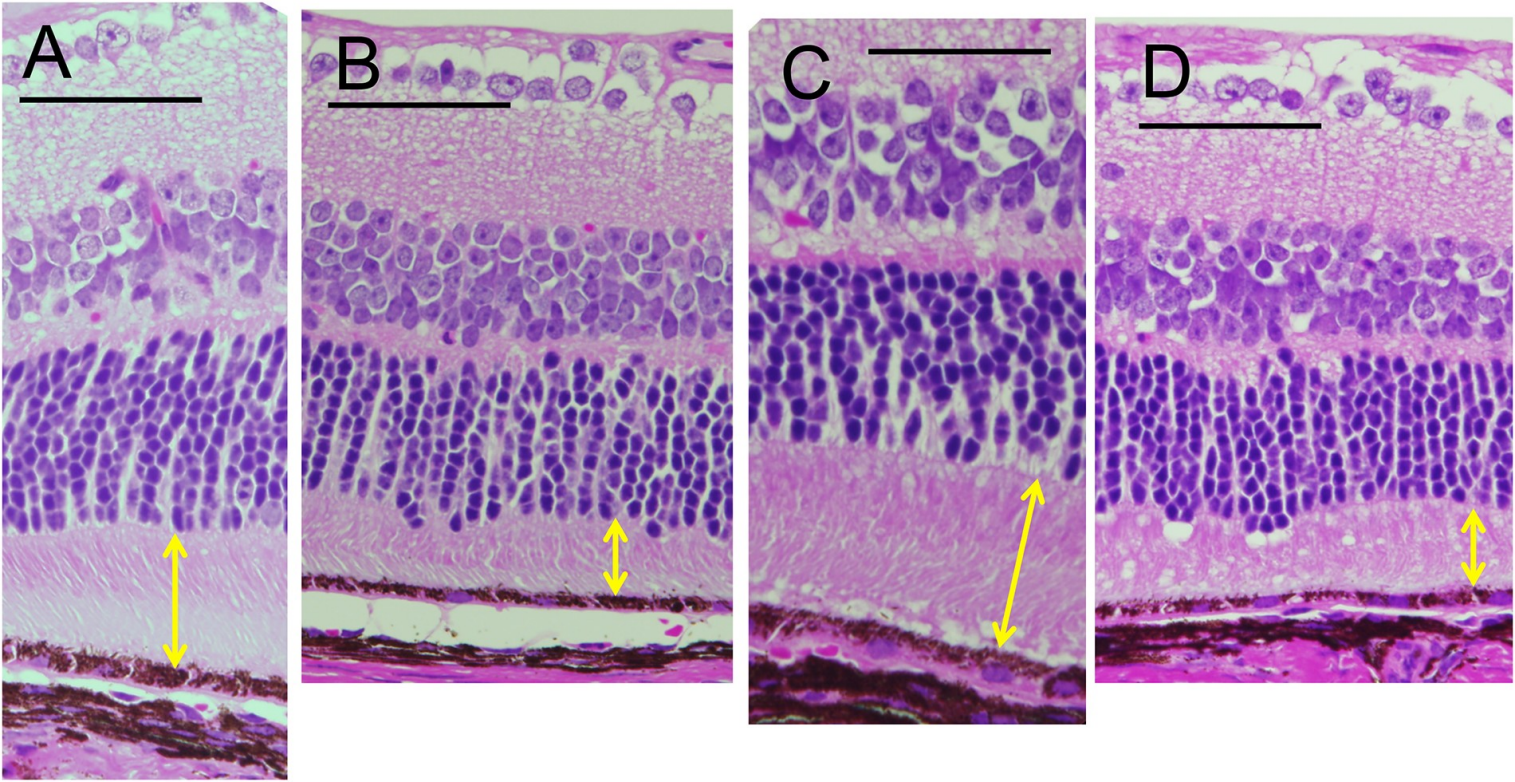
The histological findings of the C57BL/6J (A, C) and *RPE65*^−/−^ (B, D) mice (hematoxylin and eosin staining). A, A C57BL/6J mouse on P66. B, An *RPE65*^−/−^ mouse on P95. C, A C57BL/6J mouse on P120. D, An *RPE65*^−/−^ mouse on P127. Arrows indicate the thickness of the photoreceptor IS and OS layer. Bar indicates 100 μm.

**Fig 4:**
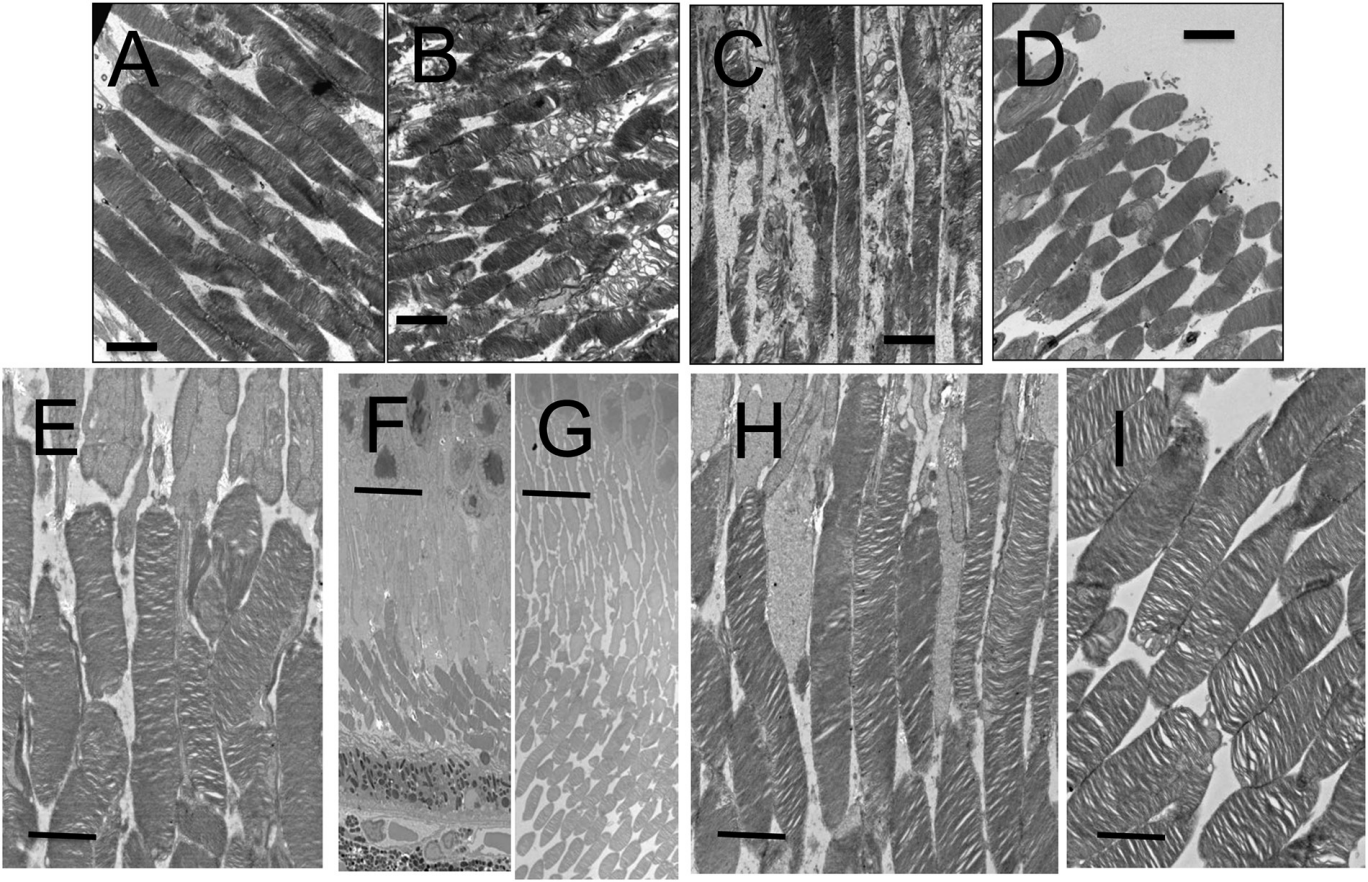
The electron microscopic findings of the C57BL/6J (A, H) and *RPE65*^−/−^ (B—G) mice. A, A C57BL/6J mouse on P21. B, An *RPE65*^−/−^ mouse on P21. C, An *RPE65*^−/−^ mouse on P35. D, An *RPE65*^−/−^ mouse on P49. E, An *RPE65*^−/−^ mouse on P95. F, An *RPE65*^−/−^ mouse on P127 at low magnification. G, A C57BL/6J mouse on P120 at low magnification. H, An *RPE65*^−/−^ mouse on P127 at high magnification. I, A C57BL/6J mouse on P120 at high magnification. Bar indicates 2 μm (A—E, H, I) or 10 μm (F, G).

We analyzed the OCT images of both C57BL/6J and *RPE65*^−/−^ mice to qualitatively characterize the OCT findings in *RPE65*^−/−^ mice. Typical OCT findings in C57BL/6J mice obtained from P22 to P168 are shown in Fig 2A, 2C, 2E, 2G and 2I. The basic structure of the retinal layers A through D (S1 Fig) appeared to be consistent throughout the observation periods. Distinct zones that appeared to correspond to the human photoreceptor inner layer ellipsoid zone (IS-EZ, Fig 2, arrows) and the interdigitation zone (IZ) were consistently observed in retinal layer C (the photoreceptor IS and OS layer, S 1) throughout the observation periods (Fig 1). Conversely, the typical OCT findings of retinal layer C in *RPE65*^−/−^ mice obtained from P22 to P170 demonstrated features that were quite different from those in C57BL/6J mice (Fig 2B, 2D, 2F, 2H and 2J). Instead of the distinct IS-EZ and IZ observed in C57BL/6J mice, retinal layer C showed a diffuse hyperreflective zone in *RPE65*^−/−^ mice (Fig 2, arrowheads), although the density of the hyperreflective zone on P22 was slightly weaker than the density on other postnatal days (Fig 2B). On comparing these findings to the histological appearance in C57BL/6J mice on P66 and 120, and *RPE65*^−/−^ mice on P95 and P127 (Fig 3), although the photoreceptor inner and outer segments were regularly arranged in C57BL/6J mice (Fig 3A and 3C), the regularity of the inner and outer segments was gradually lost, and the length of the outer segments decreased in the *RPE65*^−/−^ mice (Fig 3B and 3D, arrows). On electron microscopy, the disc structure of the photoreceptor outer segments of the *RPE65*^−/−^ mice appeared to be severely degenerated and partially vacuolated during the early postnatal period (P21 and P35, Fig 4B and 4C); however, the ratio of the degenerated outer segment discs was apparently decreased on P49 (Fig 4D) and the structure of the rod outer segment discs of the *RPE65*^−/−^ mice on P95 and P127 did not at least qualitatively appear to differ from those of the C57BL/6J mice, although the length of the outer segments of the *RPE65*^*-/-*^ mice appeared to be variable and short in comparison to those of the C57BL/6J mice (Fig 4A, 4D, 4E, 4F, 4G, 4H and 4I). These degenerative changes in the outer segments in the early postnatal period (P22 and P36) corresponded to the diffuse hyperreflective zone on OCT images (Fig 2). In addition, the variable size of the rod outer segments also corresponded to the diffuse hyperreflective zone in the OCT pictures after P79 (Fig 2). These two qualitative changes could not be differentiated on OCT images.

### The quantitative analyses of the OCT findings in *RPE65*^−/−^ mice

**Fig 5:**
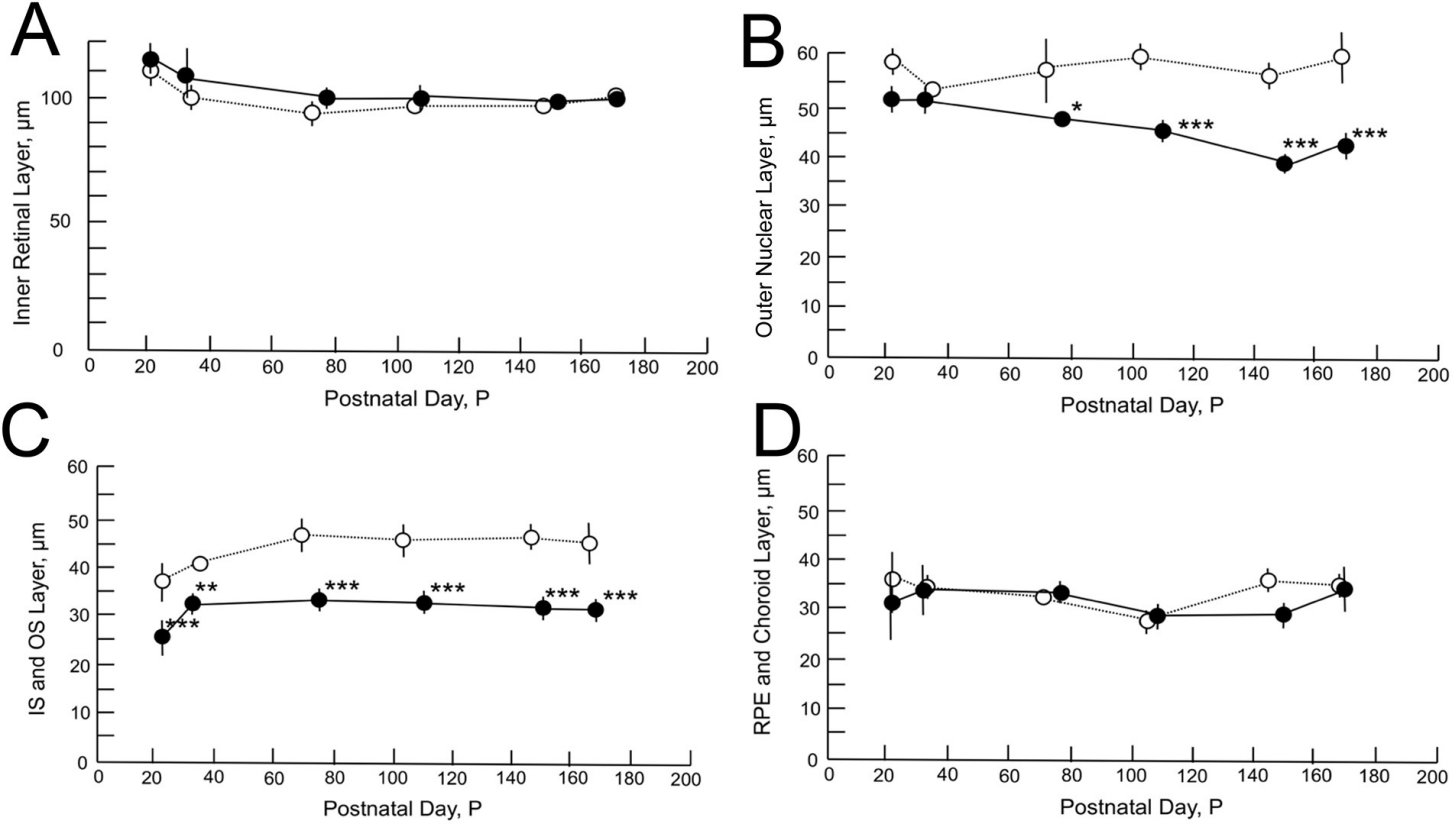
The longitudinal changes in the thicknesses of the retinal sublayers. Open circles, C57BL/6J mice; closed circles, *RPE65*^−/−^ mice. A, Thickness changes in the inner retinal layer. B, The thickness of the outer nuclear layer. C, The thickness of the photoreceptor IS and OS layer. D, The thickness of the combined RPE and choroid layer. Statistical significance: *, *P* < 0.05; **, *P* < 0.01; ***, *P* < 0.001 (Student’s *t*-test).

The longitudinal changes of the thickness of the retinal sublayer thickness are shown in Fig 5. There were no statistically significant differences in the thickness of the sublayers A and D at any time point between the *RPE65*^−/−^ and C56BL/6J mice (Fig 5A and 5D). However, there were statistically significant differences in the thickness of the sublayers B and C between two groups (Fig 5B and 5C). In retinal sublayer B, the thickness of the outer nuclear layer of the *RPE65*^−/−^ mice became significantly thinner in comparison to the C57BL/6J mice at all time points after P79 (Fig 5B). In addition, the thickness of retinal sublayer C in the *RPE65*^−/−^ mice was significantly thinner in comparison to the C57BL/6J mice at all the time points after P22 (Fig 5C), while the thickness in the *RPE65*^−/−^ mice was maintained after P36. This tendency corresponded with the results that we observed in the histological sections (Fig 3, arrows).

### The ERG findings

**Fig 6:**
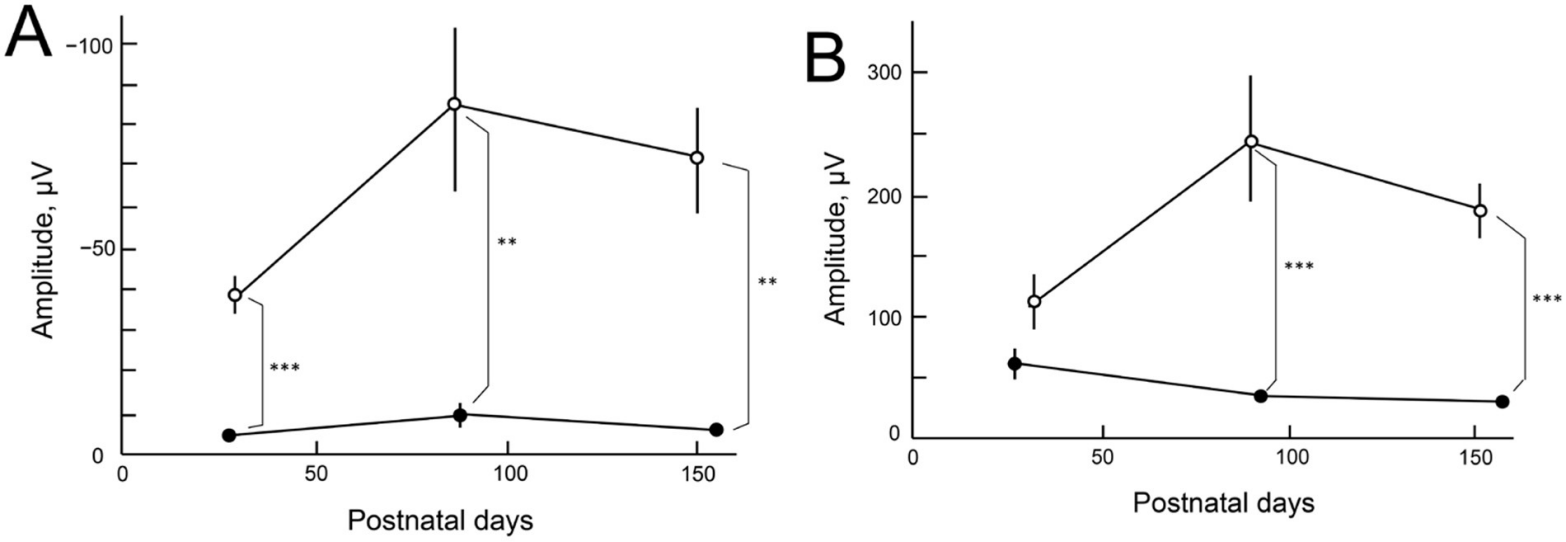
Longitudinal changes in the amplitudes of the a- and b-waves on ERG. Open circles, C57BL/6J mice; closed circles, *RPR65*^*-/-*^ mice. A, The longitudinal changes in the a-wave. B, The longitudinal changes in the b-wave. Statistical significance: **, *P* < 0.01; ***, *P* < 0.001 (Student’s *t*-test). Bars indicate standard error.

**Fig 7:**
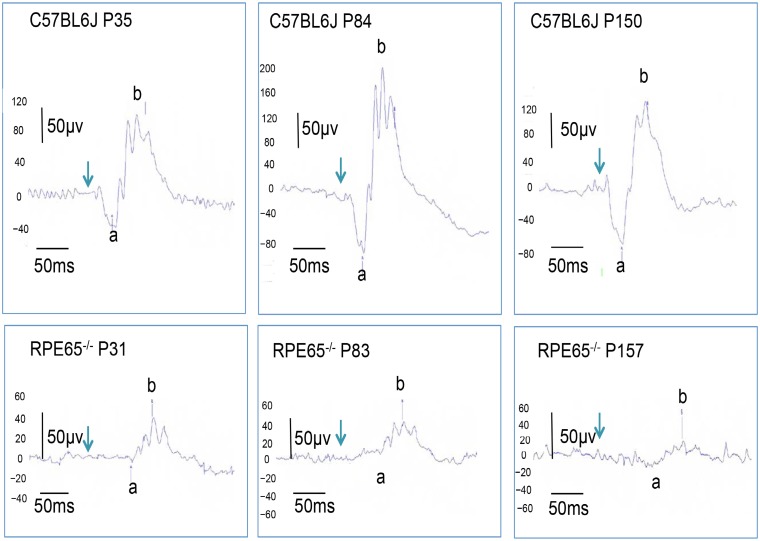
Representative ERG waveforms of the C57BL/6J and *RPR65*^*-/-*^ mice. The upper panels show ERG waveforms of the C57BL/6J on P35, P84 and P150, respectively. The lower panels show ERG waveforms of *RPR65*^*-/-*^ mice on P31, P83 and P157, respectively. Abbreviations: a, a-wave; b, b-wave.

The longitudinal changes of a- and b-waves of both *RPE65*^*-/-*^ and C57BL/6J mice are presented in Figs 6 and 7 and summarized in S3 Table. The amplitudes of the a-waves of the *RPE65*^*-/-*^ mice were severely deteriorated in comparison to those of the C57BL/6J mice at all time points measured (Fig 6, S3 Table). Similarly, the amplitudes of the b-waves of the *RPE65*^*-/-*^ mice were significantly reduced at P83 and P157 in comparison to those of the C57BL/6J mice (Fig 6, S3 Table). In addition, the latencies of both the a- and b-waves were significantly elongated in the *RPE65*^*-/-*^ mice in comparison to those in the C57BL/6J mice (S3 Table). When the a-wave latency was subtracted from the b-wave latency, there was no statistical difference between the two groups with the exception of a weak difference (*P* = 0.012) between P157 (RPE65^−/−^) vs P150 (C57BL/6J), suggesting that the elongation of the a-wave was a major cause of the total elongation of the latencies (S3 Table).

### The changes in the fundus and the corresponding OCT findings

**Fig 8:**
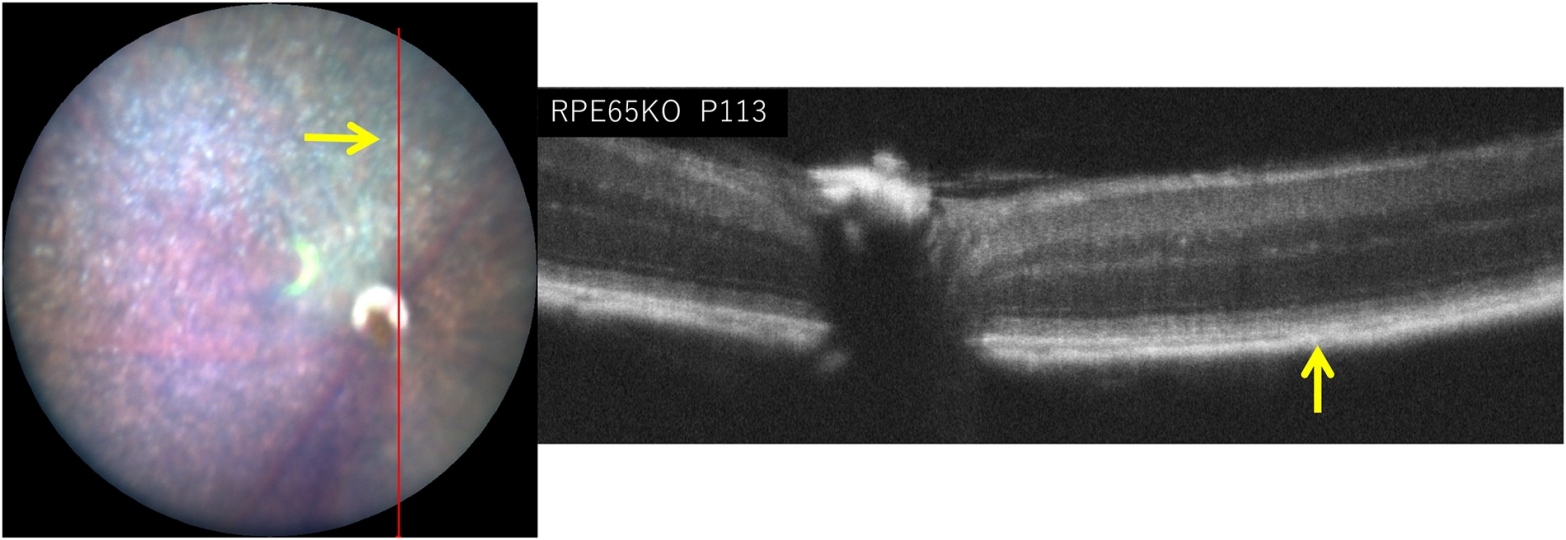
The fundus photograph (left panel) and a corresponding OCT image (right panel) of the *RPE65*^−/−^ mouse on P113. The red line in the left panel indicates the section line of the corresponding OCT image (right panel). Arrow indicates the location of a depigmented spot.

As shown in Fig 1, we found that the depigmented mottled appearance of the fundi of the *RPE65*^−/−^ mice after P79 resembled what was reported in human patients with LCA associated with *RPE65* gene mutations (Fig 1F, 1H and 1J). Thus, we observed an OCT image corresponding to the depigmented spots. Fig 8 shows a picture of the fundus and the corresponding OCT image of an *RPE65*^−/−^ mouse on P113. The arrows in Fig 8 indicate a depigmented spot (Fig 8, left) and the corresponding OCT image (Fig 8, right). The depigmented spot on the fundus appeared to correspond to a hyperreflective zone under the RPE layer.

## Discussion

In the *RPE65*^−/−^ mice, the absence of intact RPE65 results in the loss of conversion of all-trans-retinyl ester to 11-cis-retinol and the subsequent accumulation of all-trans-retinyl ester in the RPE [43]. Photoreceptor degeneration in the *RPE65*^−/−^ mice is derived from the absence of the chromophore, 11-cis-retinal, which is necessary for the structures and functions of the rod and cone opsins. The absence of 11-cis-retinal causes the mistrafficking of cone opsins to the photoreceptor outer segments [40] and downregulates the expressions of cone-specific genes [16]. As a result, the cone photoreceptors are reportedly severely degenerated from the early postnatal period in *RPE65*^−/−^ mice [15–17]. In experiments, the damages to the cone photoreceptors were rapidly restored after the administration of 9- or 11-cis retinoids, confirming the essential role of 11-cis-retinal in the cones [15–17, 43]. In contrast, despite the absence of 11-cis retinal, rod opsin proteins are trafficked to the outer segments [44] and the *RPE65*^−/−^ mice only show moderate rod degeneration [15–17, 44]. The rod photoreceptors remained long into the postnatal periods, while it was reported that the rod outer segment discs in the *RPE65*^−/−^ mice were not tightly packed in comparison to wild-type mice [15]. We previously reported the possibility that incomplete glycation of the cone opsins in the *RPE65*^−/−^ mice may result in the mislocalization of S-cone opsin and the degradation of M-cone opsin [44]. These molecular mechanisms may explain the different features of degenerations of the photoreceptor cones and rods in *RPE65*^−/−^ mice; however, further analyses are necessary to clarify the underlying mechanisms.

In the present study, we characterized the OCT findings of the retina of *RPE65*^−/−^ mice in relation to the histopathological, electron microscopic and ERG findings. Although there have been no reports of rod photoreceptor degeneration in the early postnatal periods of the *RPE65*^−/−^ mice, we observed the disarrangement and vacuolation of the discs in the photoreceptor outer segments in the early postnatal period (P21 and P35, Fig 4B and 4C). The length and width of the degenerated outer segments suggest that they were the rod photoreceptors. Interestingly, the degenerated outer segments were significantly decreased in number on P49 (Fig 4D) and the structure of the remaining outer segments appeared to resemble that of wild type mice (Fig 4E–4H), although the size of the outer segments were much shorter in comparison to wild-type mice (Fig 4F). In the OCT images of the *RPE65*^−/−^ mice, the layer of the degenerated photoreceptor IS and OS (retinal sublayer C) appeared to include diffuse hyperreflective zones (Fig 2). Considering the findings presented in Fig 4, these hyperreflective zones were derived from the disarrangement and vacuolation of the outer segment discs in the early phase and the variable sizes of the rod outer segment after 1 month of age. Both pathologic changes may cause the diffuse reflections of light and the subsequent appearance of the diffuse hyperreflective zones on OCT images. Thus, we conclude that the limitation of the current generation of OCT technology is its inability to differentiate between disorganization of the disc structure and inconsistency in size of the length of the outer segments. In contrast, one of the advantages of OCT is that we can noninvasively observe longitudinal changes in the thickness of each retinal layer. In the present study, we observed—for the first time—that the outer nuclear layer became progressively thinner, while the thickness of the photoreceptor IS and OS layer was unchanged for observational periods from P32 to P170; however, the thickness was significantly thinner in comparison to wild type mice (Fig 5). These results will be available as reference data for future animal experiments to examine possible therapeutic methods for treating retinal degeneration associated with mutations in the *RPE65* gene. For the functional aspects, the progressive and significant deficits in the amplitudes of both the a- and b-waves and the significant elongation of the latency of a-wave on ERG of the RPE65^-/-^ mice (Figs 6 and 7, S 4) indicate that the functional disturbances of the photoreceptors become severe, despite OCT features that appeared to show the relative preservation in the thickness of the photoreceptor layers (layers B and C, Fig 5B and 5C).

The fundus of the *RPE65*^−/−^ mice showed gradually progressive diffuse retinal degeneration associated with numerous depigmented spots that were distributed throughout the fundus (Fig 1). These features appeared to correspond to the appearance of the fundus in patients that was previously reported in association with compound heterozygous Leu67Arg/Tyr368Cys mutations in the RPE65 gene [41]. We attempted to observe a depigmented spot using OCT. The depigmented spot seemed to correspond to a hyperreflective zone located under the RPE layer (Fig 6), indicating that depigmented spots are derived from the abnormal changes occurring in the choroidal layer secondary to the photoreceptor degeneration. However, it is possible that these depigmented spots may be derived from various stages of RPE atrophy, which cannot be differentiated by OCT. This would be one of limitations in the OCT analysis.

The OCT images in patients with LCA associated with compound heterozygous mutations in the RPE65 gene have been reported in a previous study, which showed the photoreceptor inner and outer segment layer to be a diffuse hyperreflective zone that resembled what we observed in the *RPE65*^−/−^ mice of the present study [41]. Considering the results obtained from the present study, we hypothesize that the photoreceptor layer in patients with *RPE65* gene mutations may consist of outer segments of various sizes at the stage in which numerous depigmented spots are distributed throughout the fundus and the diffuse hyperreflective zone was seen in the outer layer of the retina on OCT.

## Supporting information

S1 Fig Definition of the retinal sublayers A, B, C and D, ELM, IS-EZ and IZ, and comparison between a representative OCT image and the histological findings. Abbreviations: NFL, nerve fiber layer; GCL, ganglion cell layer; IPL, inner plexiform layer; INL, inner nuclear layer; OPL, outer plexiform layer; ONL, outer nuclear layer; ELM, external limiting membrane; IS-EZ, inner segment ellipsoid zone; IZ, interdigitation zone; RPE, retinal pigment epithelium; IS, inner segment; OS, outer segment.
(TIF)

S1 Table Raw data for the retinal layer analysis (μm) in C57BL/6J mice. (PDF)

S2 Table Raw data for the retinal layer analysis (μm) in *RPE65*^−/−^ mice. (PDF)

S3 Table Raw data for the amplitudes and latencies of the a- and b-waves on ERG in C57BL/6J and *RPE65*^−/−^ mice. (PDF)
