# Supplementary figures and images for: The findings of optical coherence tomography of retinal degeneration in relation to the morphological and electroretinographic features in RPE65−/− mice
Source: PLoS One. 2019 Jan 29;14(1):e0210439. doi: 10.1371/journal.pone.0210439 (PMC6350961; doi:10.1371/journal.pone.0210439)

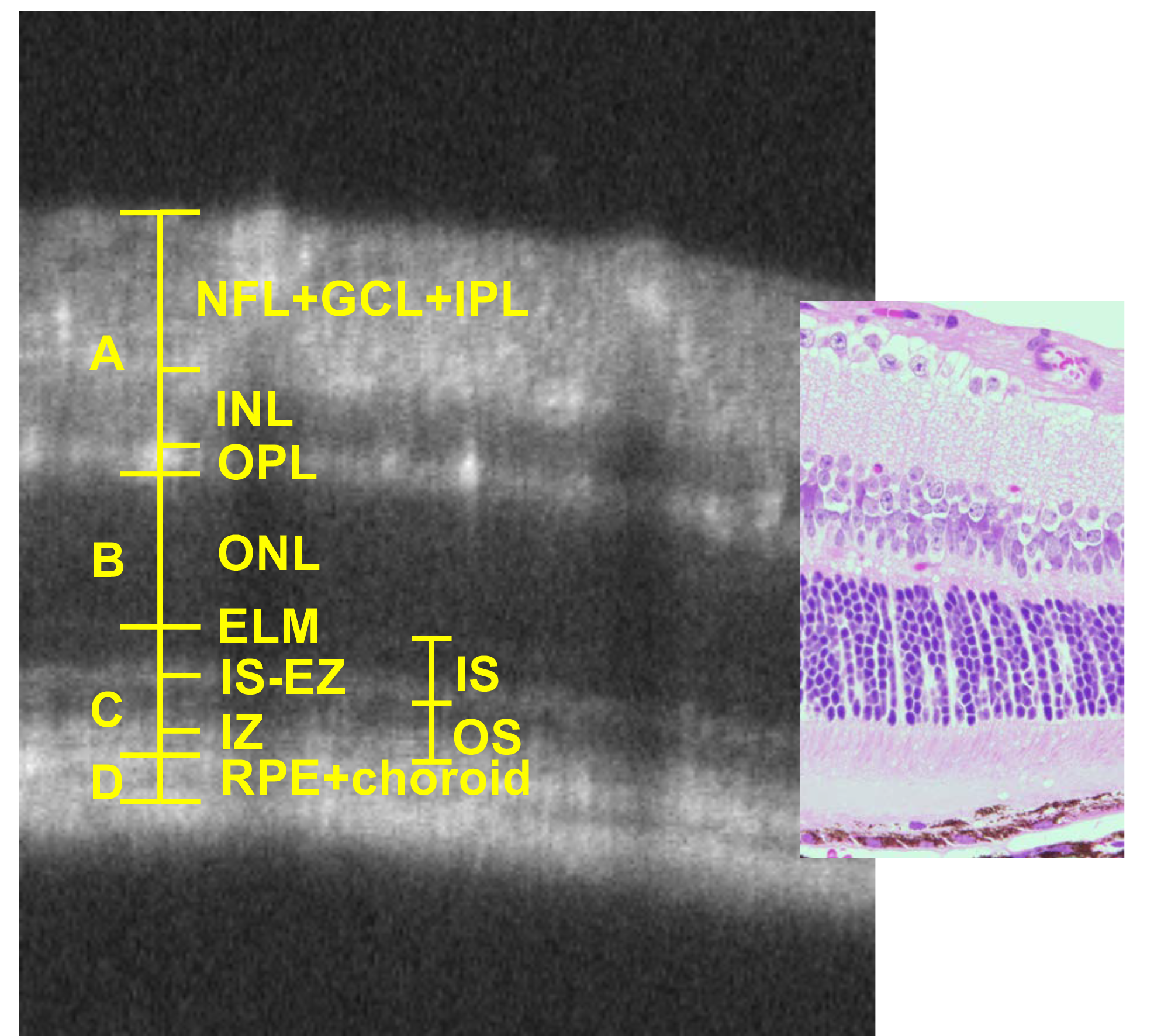

Supplement: S1 Fig — Abbreviations: NFL, nerve fiber layer; GCL, ganglion cell layer; IPL, inner plexiform layer; INL, inner nuclear layer; OPL, outer plexiform layer; ONL, outer nuclear layer; ELM, external limiting membrane; IS-EZ, inner segment ellipsoid zone; IZ, interdigitation zone; RPE, retinal pigment epithelium; IS, inner segment; OS, outer segment. (TIF) [file pone.0210439.s001.tif]
