# Supplementary material for: The findings of optical coherence tomography of retinal degeneration in relation to the morphological and electroretinographic features in RPE65−/− mice
Source: PLoS One. 2019 Jan 29;14(1):e0210439. doi: 10.1371/journal.pone.0210439 (PMC6350961; doi:10.1371/journal.pone.0210439)
Supplement: S1 Table — (PDF) [file pone.0210439.s002.pdf]

S 2 Retinal layer thickness of C57BL/6J

Age<br>nunmer	Inner Retinal Layer (A)<br>NFL, GCL, IPL, INL, OPL	Outer Retinal Layer (B)<br>ONL	IS/OS Layer (C)<br>IS, OS	RPE + Choroid (D)<br>RPE , Choroid
P22				
1	114.249	61.273	37.391	35.554
2	117.628	59.521	32.660	32.048
3	111.825	57.625	37.963	30.692
4	107.442	56.235	40.046	40.814
mean ± SD	111.717 ± 5.118	58.178 ± 2.745	37.621 ± 3.478	35.984 ± 5.923
P36				
1	105.19	54.334	40.803	34.294
2	99.195	54.814	41.388	37.548
3	98.778	55.411	41.414	32.284
mean ± SD	101.0543 ± 3.588	54.853 ± 0.540	41.202 ± 0.346	34.709 ± 2.656
P72				
1	90.362	66.758	42.737	32.991
2	93.543	55.906	48.006	31.94
3	95.923	52.056	48.262	32.002
4	99.585	53.567	49.979	31.952
mean ± SD	94.853 ± 3.891	57.072 ± 6.649	47.246 ± 3.131	32.221 ± 0.514
P106				
1	99.198	58.07	46.964	32.497
2	103.405	56.74	50.953	37.063
3	97.441	61.266	44.077	31.357
4	97.961	62.415	41.66	34.107
mean ± SD	99.5013 ± 2.705	59.622 ± 2.660	45.914 ± 3.999	28.891 ± 1.730
P148				
1	101.767	56.341	51.513	40.252
2	98.431	60.285	46.279	34.979
3	96.049	55.316	51.337	37.43
4	99.236	55.598	49.275	37.082
mean ± SD	98.871 ± 2.358	56.885 ± 2.307	49.601 ± 2.437	37.436 ± 2.167
P169				
1	99.095	61.894	42.834	37.309
2	107.032	63.628	45.519	38.889
3	102.313	54.953	51.468	34.510
4	104.480	61.984	48.965	37.200
mean ± SD	103.285 ± 2.995	59.899 ± 4.068	48.203 ± 3.824	36.603 ± 2.105
