# Supplementary material for: The findings of optical coherence tomography of retinal degeneration in relation to the morphological and electroretinographic features in RPE65−/− mice
Source: PLoS One. 2019 Jan 29;14(1):e0210439. doi: 10.1371/journal.pone.0210439 (PMC6350961; doi:10.1371/journal.pone.0210439)
Supplement: S2 Table — (PDF) [file pone.0210439.s003.pdf]

S 3 Retinal layer thickness of *RPE−/−*

Age number	Inner Retinal Layer (A) NFL, GCL, IPL, INL, OPL	Outer Retinal Leyer (B) ONL	IS/OS Layer (C) IS, OS	RPE + Chroid (D) RPE, Chroid
P22				
1	116.401	51.028	28.661	23.24
2	118.873	55.727	21.629	32.664
3	116.466	49.178	24.403	37.28
4	107.355	50.432	25.427	30.953
mean ± SD	114.774 ± 5.078	51.591 ± 2.863	25.030 ± 2.904	31.034 ± 5.843
P27				
1	107.167	52.638	33.191	49.518
2	111.603	52.495	30.144	33.536
3	112.034	49.978	28.653	37.046
4	107.45	47.063	30.121	33.564
mean ± SD	109.971 ± 2.540	50.934 ± 2.492	30.450 ± 3.332	37.440 ± 7.064
P32				
1	127.807	48.996	30.882	35.409
2	104.684	51.046	32.892	37.500
3	105.535	50.711	30.452	28.704
4	110.188	55.044	34.291	32.977
mean ± SD	110.579 ± 9.920	51.368 ± 2.236	32.282 ± 1.930	34.418 ± 3.932
P61				
1	103.669	49.462	32.080	37.506
2	99.658	50.841	34.278	32.546
3	105.481	47.297	34.767	33.888
4	105.225	47.128	34.131	33.968
mean ± SD	102.893 ± 3.60	49.172 ± 2.853	33.602 ± 2.815	34.660 ± 2.874
P79				
1	104.826	48.584	35.163	37.456
2	98.809	48.342	32.34	34.387
3	97.796	46.847	35.576	32.56
mean ± SD	100.477 ± 3.800	47.924 ± 0.941	34.360 ± 1.761	34.801 ± 2.474
P113				
1	108.787	45.76	35.085	29.117
2	101.523	44.793	35.228	30.901
3	96.429	43.802	36.765	26.68
4	99.931	48.982	32.048	28.867
mean ± SD	101.668 ± 5.201	45.834 ± 2.246	34.782 ± 1.975	28.891 ± 1.730
P155				
1	96.122	38.883	31.512	29.68
2	101.275	41.354	30.736	30.009
3	100.323	38.439	35.548	35.527
mean ± SD	99.240 ± 2.742	39.559 ± 1.571	32.599 ± 2.584	31.739 ± 3.285
P170				
1	101.315	44.033	31.795	34.588
2	101.086	43.512	28.555	38.013
3	100.412	40.171	32.756	31.85
4	102.436	39.851	32.901	30.953
mean ± SD	101.275 ± 2.270	42.519 ± 2.222	31.059 ± 2.098	'34.667 ± 4.459
