# Supplementary material for: The findings of optical coherence tomography of retinal degeneration in relation to the morphological and electroretinographic features in RPE65−/− mice
Source: PLoS One. 2019 Jan 29;14(1):e0210439. doi: 10.1371/journal.pone.0210439 (PMC6350961; doi:10.1371/journal.pone.0210439)
Supplement: S3 Table — (PDF) [file pone.0210439.s004.pdf]

S 4 ERG summary

Mouse<br>Age and number	a−wave amplitude<br>μV	a−wave latency<br>ms	b−wave amplitude<br>μV	b−wave latency<br>ms	(b−a) latency<br>ms
C57BL6J					
P35					
1	−43.0	22.06	149.5	58.82	36.76
2	−42.0	30.88	117.00	60.29	29.41
3	−30.0	33.82	70.00	69.12	35.30
mean ± SE	−38.33 ± 4.18	28.92 ± 3.53	112.17 ± 23.08	62.74 ± 3.22	33.82 ± 2.25
P84					
1	−75.50	22.77	252.75	48.49	25.69
2	126	25.00	331.34	51.47	26.47
3	−23.00	25.00	68.00	51.47	26.47
mean ± SE	−85.20 ± 22.87	24.12 ± 0.75	247.23 ± 52.85	50.28 ± 1.28	26.26 ± 0.74
P150					
1	−37.50	24.26	124.50	56.52	32.36
2	−85.00	31.62	190.00	60.29	28.68
3	−80.00	30.88	199.00	60.29	29.41
4	−107.50	27.94	270.00	58.82	30.88
mean ± SE	−72.08 ± 13.05	28.43 ± 1.45	183.00 ± 26.64	58.82 ± 2.41	30.39 ± 2.39
RPE65KO					
P31					
1	−5.00	41.18	74.23	70.59	29.41
2	−5.00	50.00	50.00	82.35	32.35
3	−6.00	38.24	79.5	76.47	38.23
4	−6.50	39.70	30.00	86.76	47.06
mean ± SE	−5.63 ± 0.375***	42.28 ± 2.64*	58.43 ± 11.45	79.04 ± 3.52*	36.76 ± 3.89
P83					
1	−24.50	47.79	29.50	78.68	30.89
2	−5.00	62.50	31.50	84.93	22.44
3	−5.00	58.09	29.00	90.45	32.36
4	−8.50	60.30	38.50	100.00	39.70
mean ± SE	−10.75 ± 3.07**	57.17 ± 3.36***	32.13 ± 2.55***	88.51 ± 3.69***	31.35 ± 2.65
P157					
1	−5.00	61.76	31.00	104.41	42.65
2	−7.50	55.15	27.00	99.27	45.12
3	−6.50	54.41	19.50	105.15	50.74
4	−10.00	48.52	17.00	117.65	69.13
mean ± SE	−6.50 ± 1.03**	56.17 ± 2.10***	24.80 ± 2.37***	102.65 ± 3.89***	46.47 ± 4.91*

Statistical significance: ***, $P < 0.001$; **, $P < 0.01$; *, $P < 0.05$

SE, standard error
